# Ion Channel Dysfunction in Astrocytes in Neurodegenerative Diseases

**DOI:** 10.3389/fphys.2022.814285

**Published:** 2022-02-09

**Authors:** Sijian Wang, Biyao Wang, Dehao Shang, Kaige Zhang, Xu Yan, Xinwen Zhang

**Affiliations:** ^1^Center of Implant Dentistry, School and Hospital of Stomatology, China Medical University, Liaoning Provincial Key Laboratory of Oral Diseases, Shenyang, China; ^2^The VIP Department, School and Hospital of Stomatology, China Medical University, Liaoning Provincial Key Laboratory of Oral Diseases, Shenyang, China

**Keywords:** astrocytes, aquaporins, transient receptor potential channel, ATP-sensitive potassium channel, inwardly rectifying potassium channel, KCa3.1 channel, P2X7 receptor, neurodegenerative disease

## Abstract

Astrocytes play an important role in the central nervous system (CNS). Ion channels in these cells not only function in ion transport, and maintain water/ion metabolism homeostasis, but also participate in physiological processes of neurons and glial cells by regulating signaling pathways. Increasing evidence indicates the ion channel proteins of astrocytes, such as aquaporins (AQPs), transient receptor potential (TRP) channels, adenosine triphosphate (ATP)-sensitive potassium (K-ATP) channels, and P2X7 receptors (P2X7R), are strongly associated with oxidative stress, neuroinflammation and characteristic proteins in neurodegenerative disorders, including Alzheimer’s disease (AD), Parkinson’s disease (PD), Huntington’s disease (HD) and amyotrophic lateral sclerosis (ALS). Since ion channel protein dysfunction is a significant pathological feature of astrocytes in neurodegenerative diseases, we discuss these critical proteins and their signaling pathways in order to understand the underlying molecular mechanisms, which may yield new therapeutic targets for neurodegenerative disorders.

## Introduction

Astrocytes are the most abundant cells in the central nervous system (CNS). These cells are capable of transporting ions, taking up neurotransmitters and producing neurotrophic factors to maintain the function and homeostasis of the CNS, where astrocytic ion channels have a pivotal role (Ikeshima-Kataoka, 2016; Verkhratsky and Nedergaard, 2018). Transient receptor potential (TRP) channels are a family of calcium ion (Ca^2+^) channels responsible for the transport of Ca^2+^ in the plasma membrane and endoplasmic reticulum (ER), maintaining astrocyte Ca^2+^ homeostasis and dynamics (Zhang and Liao, 2015; Lim et al., 2016; Schmaul et al., 2021). The sensitivity of KCa3.1 channels to Ca^2+^ means they are capable of transporting potassium ion (K^+^) in response to Ca^2+^ fluctuations (Yi et al., 2017). Inwardly rectifying K^+^ (Kir) and adenosine triphosphate (ATP)-sensitive K^+^ (K-ATP) channels consisting of Kir channel isoforms on astrocyte terminal protrusions (also known as end-feet) participate in extracellular K^+^ transport and mediate mitochondrial autophagy as well as glutamate uptake (Tinker et al., 2018; Kinboshi et al., 2020). Water channel protein aquaporin (AQP) 4 channels are located on astrocyte terminal protrusions, and mediate transfer between cerebrospinal fluid (CSF) and intercellular fluid (Iliff et al., 2012). In addition, astrocytes have a multi-ion transport channel P2X7 receptor (P2X7R), which is sensitive to ATP and permeable to sodium ion (Na^+^), K^+^ and Ca^2+^ (Zhao et al., 2021). In conclusion, these ion channels regulate astrocytic function by moderating ion homeostasis in astrocytes, while their homeostatic role on extracellular ions and related derivative effects maintain the normal physiological status of the CNS.

However, abnormalities in astrocytic ion channel expression, localization, and function can cause disruption of ion homeostasis and neurotransmitters, deposition of characteristic proteins, oxidative stress, and neuroinflammation. When astrocytic AQPs are abnormally located or down-regulated, the flow of brain interstitial solution mediated by AQPs is reduced, and, subsequently, pathological markers such as amyloid-β (Aβ), tau, and mutant α-synuclein (α-SYN) accumulate due to a failure of clearance (Zou W. et al., 2019; Harrison et al., 2020). These substances trigger the production of reactive oxygen species (ROS) and inflammatory factors, which can activate TRP channels leading to astrocytic Ca^2+^ overload *via* excessive Ca^2+^ influx and ER Ca^2+^ depletion (Yamamoto et al., 2007; Bosson et al., 2017; Lee et al., 2019). Excessive Ca^2+^ not only causes astrogliosis, but also induces KCa3.1 channels to generate more pro-inflammatory factors (Bouhy et al., 2011). The ionic disturbance of astrocytes is not only reflected in the above channels, but P2X7R can also cause an imbalance of Ca^2+^ and K^+^, resulting in overactivation of astrocytes (Zhao et al., 2021). What is more, deficiencies in K-ATP and Kir channels not only cause an imbalance of K^+^, but a loss of their protective effects leads to astrocyte swelling as a result of the decreased permeability of gap junctions (Bataveljić et al., 2012; Tinker et al., 2018). Meanwhile, K-ATP channel deficiencies can trigger the aggregation of damaged mitochondria caused by impaired mitophagy and the activation of inflammatory pathways, resulting in astrocyte activation and neuroinflammation (Hu et al., 2019; Chen et al., 2021). Therefore, aberrant astrocytic ion channels appear to be a major link mediating a vicious cycle of astrocyte abnormalities and nervous system injuries.

In this paper, we will summarize the physiological role played by astrocytic ion channels in the CNS and the link between abnormalities of such ion channels and neurological disorders. Thereafter, we will focus on the pathogenesis of several common neurodegenerative diseases and specifically discuss the role of ion channels involved in these disorders and the advances in research related to the treatment of these diseases.

## Ion Channels in Astrocytes

Aquaporins are a class of water channel proteins involved in the transport of water together with a number of small molecules, including glycerin, urea, and H_2_O_2_. To date, thirteen members of AQPs (AQP0 to 12) have been identified in mammals. Of these, AQP4 is the most expressed isoform in the human CNS. In the brain, AQP4 is widely distributed in astrocyte terminal protrusions where it takes part in lymphatic drainage and is closely associated with astrocyte function and homeostasis (Yang et al., 2016; Abir-Awan et al., 2019; Tamtaji et al., 2019). It was found that AQP4 mediates the exchange of arterial paracellular CSF with interstitial fluid (ISF) in the interstitial extracellular space, which facilitates the movement of interstitial solutes from brain parenchyma to CSF. The clearance of interstitial solutes from the brain was reduced by approximately 70% in AQP4-deficient animals (Iliff et al., 2012). In addition, the astrocytic AQP4 channel is involved in cellular edema in the CNS. Its ability to mediate water clearance in vasogenic edema is accompanied by a concomitant exacerbation of intracellular edema. The alleviating effect of knocking out AQP4 on brain edema in animal models has been shown (Papadopoulos et al., 2004; Kitchen et al., 2020). In addition, the overexpression and altered subcellular localization of AQP4 channels in astrocytes has been suggested as a reason for blood–brain barrier (BBB) dysfunction, which contributes to the pathological progression of neurodegenerative diseases (Amiry-Moghaddam et al., 2003; Tang et al., 2014; Glober et al., 2019).

Transient receptor potential channels are a group of Ca^2+^ channels that are distributed in neurons, glial cells, and cerebral vascular endothelium. In the CNS, TRP channels can be activated by a variety of physical and chemical stimuli, thus participating in various physiological and pathological processes such as cell proliferation, activation, osmoregulation and oxidative stress (Zhang and Liao, 2015; Echeverry et al., 2016). According to amino acid sequence homology, seven isoforms have been identified, including TRPA (TRP ankyrin), TRPC (canonical), TRPM (melastatin), TRPML (mucolipin), TRPN (no mechanopotential), TRPP (polycystin), and TRPV (vanilloid) channels. Of these, except for TRPN, the other six are expressed in mammals (Zhang and Liao, 2015). Astrocytes are Ca^2+^ excitable and the influx of extracellular Ca^2+^ and release of Ca^2+^ from ER increase the concentration of Ca^2+^ in the cytoplasm of astrocytes. In astrocytes, TRP channels are able to mediate Ca^2+^ influx across the cell membrane and participate in store-operated Ca^2+^ channel (SOC)–mediated Ca^2+^ to enter the ER, thereby maintaining intracellular Ca^2+^ homeostasis (Zhang and Liao, 2015; Lee et al., 2016; Lim et al., 2016). However, in pathological states, TRP channels in astrocytes can be activated by ROS, nitric oxide (NO), inflammatory factors, and pathological markers of neurodegenerative diseases, such as Aβ, which disrupts Ca^2+^ homeostasis. Astrocytic Ca^2+^ overload can cause excessive activation of astrocytes, and reactive astrocytes can lead to neurodegeneration by releasing ROS, reactive nitrogen species (RNS), and pro-inflammatory factors (Yamamoto et al., 2007; Bosson et al., 2017; Lee et al., 2019).

The influx of Ca^2+^ into cells can elicit a response from Ca^2+^-sensitive channels, which typically include Ca^2+^-activated K^+^ (KCa) channels. KCa3.1 is one such channel and belongs to the intermediate conductance Ca^2+^-activated K^+^ channel family according to a conductance classification system (Faber and Sah, 2003; Sugunan et al., 2016). Under physiological conditions, KCa3.1 channels are able to participate in the regulation of membrane potential by controlling the passage of K^+^ in response to the inward flow of Ca^2+^ (Yi et al., 2017). Meanwhile, gliosis and cell death caused by intracellular Ca^2+^ overload are also involved in KCa3.1 channel activity. During astrocyte activation, KCa3.1 channels can up-regulate pro-inflammatory factors, including interleukin (IL)-1β and tumor necrosis factor (TNF)-α, which lead to neuroinflammation (Bouhy et al., 2011). In addition, KCa3.1 also engages in astrocytic ER stress through excessive store-operated Ca^2+^ entry (SOCE) due to an activation effect on Ca^2+^ release–activated Ca^2+^ channel protein 1 (Orai1) (Yu et al., 2018). The regulation of KCa3.1 channels also showed therapeutic effects. Evidence exists that inhibiting or blocking KCa3.1 channels can reduce reactive astrogliosis and astrogliosis-induced neuroinflammation, which is relevant to the treatment of neurological disorders including neurodegenerative diseases (Sugunan et al., 2016; Yi et al., 2016, 2017).

K-ATP channels allow the passage of K^+^ when ATP levels decrease, thus hyperpolarizing the membranes of neurons, astrocytes, microglia, and the vascular system in the CNS. Such channels are composed of Kir6.1, Kir6.2 pore-forming subunits, and sulfonylurea receptors, including SUR1, SUR2A, and SUR2B. Glutamate is an excitatory neurotransmitter involved in many physiological functions in the CNS. Nevertheless, high extracellular glutamate levels can cause excitotoxicity. Under the circumstances, K-ATP channels in astrocytes are associated with glutamate uptake and gap junctions, controlling glutamate concentrations below neurotoxic levels as well as maintaining gap junction intercellular communication, respectively, thereby playing important roles in the maintenance of normal CNS function (Zingman et al., 2007; Sun and Hu, 2010; Tinker et al., 2018).

The Kir6.1 and 6.2 subunits of K-ATP channels are both members of the Kir channel family (Sun and Hu, 2010). Kir channels are a series of tetramers that mediate the entry of K^+^, which are present in neurons and glial cells in the CNS, participating in the maintenance of extracellular K^+^ homeostasis, and this is related to the survival of neurons (Hibino et al., 2010; Kinboshi et al., 2020). Kir channels are made up of seven members (Kir1.x to Kir7.x). In addition to Kir6.x, Kir4.1 is also abundant in astrocytes. As a K^+^-transport channel subtype of the Kir channel family, Kir4.1 channels participate in astrocyte-mediated K^+^ buffering, manifesting as an uptake of excess extracellular K^+^ and translocation of K^+^ into the capillaries in a process known as spatial K^+^ buffering (Ohno et al., 2018). This process involves the participation of glutamate transporters. A deficiency of Kir4.1 can cause depolarization of the astrocytic membrane that can subsequently lead to impairment of excitatory amino acid transporters, thereby affecting the clearance of glutamate (Kinboshi et al., 2020). Glutamate excitotoxicity has been linked to neurodegenerative diseases. Meanwhile, animal models and postmortem studies of these diseases have also demonstrated a reduction in Kir4.1 channels. Therefore, Kir4.1 channels have also become a research hotspot in the study of neurodegenerative diseases (Nwaobi et al., 2016).

P2X7R is one of seven members (P2X1R∼7R) of the ligand-gated cationic channel P2X receptor (P2XR) family (Miras-Portugal et al., 2017). As an ATP-gated non-selective cationic channel with three subunits, P2X7R allows Ca^2+^, K^+^, and Na^+^ to pass through the plasma membrane, and is also involved in neuroinflammation. In microglia, P2X7R is involved in the secretion of NOD-, LRR- and pyrin domain-containing protein 3 (NLRP3) inflammatory vesicles (Crabbé et al., 2019; Illes, 2020). However, astrocytes lack NLRP3 and thus activation of their P2X7R cannot result in an NLRP3-mediated immunoreaction. Despite the lower density of P2X7R in astrocytes compared to microglia, coupled with astrocytic NLRP3 deficiency, P2X7R-mediated Ca^2+^ influx and K^+^ efflux can still activate astrocytes, resulting in the release of glutamate and inflammatory factors that cause excitotoxicity and neurotoxicity (Illes, 2020; Zhao et al., 2021).

## Astrocytic ion Channels in Alzheimer’s Disease

### Astrocytic Aquaporin 4 in Alzheimer’s Disease

In Alzheimer’s disease (AD), CSF–ISF exchange mediated by AQP4 is one of the ways in which the disease’s pathological markers are cleared (Harrison et al., 2020). Aβ is considered a pathological marker of AD and its imbalance in production and clearance is a significant feature of AD (Lee et al., 2017). For Aβ clearance, it was found that soluble Aβ was cleared by AQP4-dependent ISF bulk flow along the gliovascular clearance system, while in the CSF of AQP4-deficient mice, Aβ clearance showed a significant decrease (Iliff et al., 2012). In addition, an impaired glia–lymphatic system in patients with AD also triggered the failure of CSF–ISF exchange, and the deposition of Aβ and cerebral metabolic waste due to the mislocalization of astrocyte AQP4 (Reeves et al., 2020).

Regarding the effect of AD pathological markers on AQP4, a study with multiple transgenic mouse models of Aβ deposition indicated that the number of astrocyte terminal protrusions and localized AQP4 were significantly reduced in mice that developed cerebral amyloid angiopathy (CAA; associated with the accumulation of Aβ in the brain vasculature, a disease present in the majority of patients with AD) (Wilcock et al., 2009). However, expression of AQP4 was not decreased at mRNA and protein levels, suggesting that AQP4 exhibits aberrant localization rather than downregulation in AD. When discussing a reason for the abnormal localization of AQP4, current evidence favors downregulation of DP71 dystrophin (Wilcock et al., 2009). As an anchoring protein for AQP4 and several ion channels, DP71 dystrophin is also located in terminal protrusions of astrocytes (Eide and Hansson, 2018). The above study also found that the expression of DP71 dystrophin was downregulated in mice in the CAA group. Thus, the downregulation of DP71 dystrophin in astrocytes may contribute to the mislocalization of AQP4 in AD (Wilcock et al., 2009).

In addition to Aβ, the microtubule-associated protein tau is also considered a neuropathological hallmark of AD. In AD, hyperphosphorylation increases abnormal aggregation of tau resulting in neurogenic fibrillary tangles that contribute to synaptic dysfunction, mitochondrial impairment, and oxidative stress. Similar to Aβ, tau can diffuse and aggregate between neurons *via* the ISF (Gao et al., 2018). With regard to the involvement of AQP4 in the glia–lymphatic clearance of tau in the brain, inhibition of AQP4 in mice leads to dysfunction of CSF–ISF exchange and a significant decrease in tau clearance (Harrison et al., 2020). The same presentation and a more severe neurogenic fibrillary pathology were found in AQP4 knockout (KO) mice (Iliff et al., 2014). In addition to AQP4 channels on astrocytic terminal protrusions, AQP4 on astrocytic cell membranes near neurons also exerts an important role in the clearance of tau in mice although these AQP4 channels exist in smaller amounts (Nagelhus and Ottersen, 2013; Wu et al., 2021). Increasing AQP4 levels on astrocytic perivascular terminal protrusions did not restore the impairment of tau clearance in IL-33–deficient mice that lacked AQP4 on the astrocytic cell membrane in close proximity to neurons (Wu et al., 2021). Moreover, a postmortem study has shown that deficiency of AQP4 localization in cerebral perivascular astrocytes enlarged the perivascular space and triggered further aggregation of tau in patients with AD (Boespflug et al., 2018). Above all, dysfunction of glymphatic clearance due to a AQP4 positioning anomaly in AD highly correlates with tau aggregation and subsequent neurodegeneration.

### Astrocytic Transient Receptor Potential Channels in Alzheimer’s Disease

The TRPA1 channel is the only subtype of TRPA channel that exists in mammals. As a non-selective Ca^2+^ channel, TRPA1 channels allow Ca^2+^ and Zn^2+^ to pass through the plasma membrane. In the CNS, TRPA1 channels maintain the normal physiological functions of astrocytes and regulate brain development. Additionally, they are capable of sensing ROS and several toxic stimuli before undergoing activation. The activation of TRPA1 channels can cause headache symptoms, and neurodegenerative diseases such as AD (Zhang and Liao, 2015; Lee et al., 2016). Lee et al. (2016) found that the expression of TRPA1 was significantly higher in astrocytes of Aβ precursor protein (APP)/PS1 Tg mice than in that of controls. More specifically, this study showed that Aβ triggers TRPA1 to induce Ca^2+^ inward flow in astrocytes, thereby activating nuclear factor (NF)-κB, serine/threonine-protein phosphatase 2B (PP2B), and nuclear factor of activated T-cells (NFAT), which resulted in inflammatory responses. In addition, the Aβ oligomer–mediated Ca^2+^ influx that promotes astrocyte hyperactivation *via* activation of TRPA1 can also lead to neuronal hyperactivation, while this characteristic of astrocytes is independent of microglia and neurons. This process is also marked in the early stages of the disease in APP/PS1-21 mice, suggesting that astrocyte hyperactivity might be responsible for abnormal neuronal function in the early stages of AD (Bosson et al., 2017). Recently, intraperitoneal injection of the TRPA1 inhibitor, HC030031, in mice was found to effectively alleviate Aβ-induced astrocyte hyperactivation in the hippocampus; meanwhile, neuronal activity was also modulated. The integrity of synapses and mouse behavior also improved. Since the drug can be delivered across the BBB to the brain, its inhibition of TRPA1 is a potential pharmacological therapeutic target for AD (Paumier et al., 2021).

The effect of Aβ on Ca^2+^ influx–activated astrocytes is not limited or based on the direct stimulation of TRPA1. In fact, the activating effect of Aβ on TRPC channels also promotes SOCE-mediated Ca^2+^ influx (Yamamoto et al., 2007). Of these, astrocytic TRPC1 channels interact with Orai1 and stromal interacting molecule 1 (STIM1), and are jointly involved in SOCE, which drives sustained and oscillating Ca^2+^ signals in astrocytes (Linde et al., 2011; Lu et al., 2017). Specifically, when Ca^2+^ in the ER is depleted, STIM1 on the ER membrane recruits TRPC1-trafficking vesicles anchoring to the cell membrane; then SOC channels, consisting of STIM1–TRPC1 complexes, facilitate Ca^2+^ entry into the ER. Meanwhile, STIM1 and Orai1 that form Ca^2+^ release–activated Ca^2+^ (CRAC) channels also mediate Ca^2+^ influx (Ong et al., 2016; Kwon et al., 2017). Aβ can enhance the SOCE of 3 × Tg-AD mouse astrocytes by upregulating the TRPC1 gene. However, lipopolysaccharide (LPS) and inflammatory factors, including IL-1β and TNF-α, can down-regulate TRPC1 expression in mice astrocytes, showing the opposite effect to Aβ on SOCE (Ronco et al., 2014). TRPC1 and Orai1 were downregulated in astrocytes of APP KO mice that exhibited decreased SOCE, reaffirming the facilitative effect of Aβ on SOCE (Linde et al., 2011). Therefore, the upregulation of astrocytic TRPC1 and Orai1 by Aβ, and the resulting promoting effects on SOCE, may be new directions for the study of AD.

Additionally, TRPC6 exhibits protective effects on astrocytes including inhibiting astrocyte Ca^2+^ hyperconcentration and suppressing the astrocytic inflammatory response (Liu et al., 2020). In AD, TRPC6 has a negative effect on the production of Aβ, thereby reducing its level. In addition, a study found that TRPC6 mRNA levels were decreased in the hemocytes of patients with AD (Lu et al., 2018). Therefore, the specific role of TRPC6 in astrocytes in AD requires further research and its neuroprotective role suggests this is a possible therapeutic target for AD.

As a member of the TRPV family, TRPV4 channels are highly permeable to Ca^2+^ and are also sensitive to oxidative stress and inflammatory stimuli (Yamamoto et al., 2007; Bai and Lipski, 2014). TRPV4 channels in AD can be activated by Aβ. In disassociated rat hippocampal cultures, Aβ_40_ upregulates TRPV4 and glial fibrillary acidic protein (GFAP) expression in astrocytes, accompanied by an increase in astrocyte Ca^2+^ levels, ROS production, and significant neuronal damage, while inhibition of TRPV4 effectively reduced cell death (Bai and Lipski, 2014). In addition to TRPV4, TRPV1 in astrocytes is also capable of being activated by Aβ. Levels of TRPV1 were upregulated and those of peroxisome proliferator-activated receptor (PPAR)-α and PPAR-γ were decreased in fatty acid amide hydrolase-KO astrocytes in the presence of Aβ_1–42_, elevating levels of IL-1β, TNF-α, inducible nitric oxide synthase (iNOS), and cyclooxygenase (COX) 2 (Benito et al., 2012). Moreover, an *in vitro* study of human astrocytes revealed that TRPV1-mediated Ca^2+^ inward flow can activate c-Jun N-terminal kinase (JNK) and thus induce protective autophagy in astrocytes (Liu et al., 2013). Evodiamine, a Chinese herbal medicine for AD, can promote these processes, while inhibition of TRPV1 suppresses autophagy and triggers apoptosis in astrocytes under hypoxic conditions (Liu et al., 2013). In pathological states, autophagy is neuroprotective due to its ability to remove functionally abnormal structures and aggregated proteins, reducing their impact on astrocytes (Liu et al., 2013; Luo et al., 2020). Thus, the increase in astrocyte Ca^2+^ levels by TRPV1 and subsequent autophagy may be protective against neuronal damage in AD.

### Astrocytic KCa3.1 Channels in Alzheimer’s Disease

The role of elevated Ca^2+^ concentrations in astrocytes is not only reflected as mentioned above. Of these, the excessive activation of astrocytes caused by Ca^2+^ overload and neuroinflammation mediated by astrogliosis also includes the participation of KCa3.1 channels (Yi et al., 2016, 2017). Yi et al. (2016) found that KCa3.1 expression was upregulated in reactive astrocytes and neurons in both patients with AD and senescence-accelerated mouse prone 8 models. Inhibition or knockdown of KCa3.1 significantly inhibited Aβ oligomer–mediated Ca^2+^ inward flow into astrocytes, and astrogliosis and memory loss in mice. Moreover, KCa3.1 is capable of activating Orai1 and enhancing the STIM1-Orai1 complex–mediated CRAC current, thereby inducing excessive SOCE that leads to astrocytic ER stress and astrocytic hyperactivation. Knockdown or inhibition of the KCa3.1 channel not only effectively inhibited the ER stress caused by the above process, but also activated the protein kinase B (AKT)/mammalian target of rapamycin (mTOR) pathway by regulating Ca^2+^ concentration and ER stress, thereby protecting neurons from neurotoxicity caused by astrogliosis (Yu et al., 2018). Furthermore, Wei et al. (2019) also indicated that ER stress and Ca^2+^ overload was induced by activation of KCa3.1 on Orai1 in LPS-treated mice and cultured astrocytes *in vitro*. It was also found that KCa3.1 indirectly caused astrogliosis, neural damage, and higher tau phosphorylation through phosphatidylinositol 3-kinase (PI3K)/AKT/glycogen synthase kinase-3β (GSK-3β) and NF-κB pathways. In addition, the c-Jun/JNK pathway has also been shown to be one of the pathways enabling KCa3.1 to activate astrocytes. In astrocytes under oxygen–glucose deprivation, inhibition or deletion of KCa3.1 down-regulated the expression of JNK and extracellular-signal-regulated kinase (ERK1/2) pathways and alleviated ER stress (Yu et al., 2017). The above results fully reflect the important role of KCa3.1 and CRAC channels in ER stress in astrocyte proliferation and subsequent neurodegeneration. These pathways not only enrich the specific mechanism of KCa3.1 in AD, but also further suggest a potential role for KCa3.1 in this disease and even the reduction of Ca^2+^ overload in astrocytes for the treatment of AD.

### Astrocytic ATP-Sensitive Potassium and Kir Channels in Alzheimer’s Disease

Excessive intercellular glutamate can cause excitotoxicity, which is no exception in AD (Hynd et al., 2004). Astrocytic K-ATP channels can promote the uptake of glutamate by astrocytes therefore reducing the level in order to exhibit neuroprotective effects (Tinker et al., 2018). However, abnormalities in K-ATP channels, including their Kir subunits, contribute to the pathogenesis of AD. Specifically, Aβ_1–42_ increased the expression of neuronal K-ATP channels (Bláhová and Bébarová, 2021). The induction of K-ATP opening by diazoxide effectively controlled oxidative stress and reduced levels of Aβ oligomers and hyperphosphorylated tau in the hippocampus and cortex, improving behavioral performance in 3 × Tg-AD mice (Liu et al., 2010). Griffith et al. (2016) reported that Kir6.2 subunits were significantly increased in reactive astrocytes in both patients with AD and the hippocampus in 3 × Tg-AD mice. Since excitatory amino acid transporter 2 is reduced in AD in order to promote glutamate uptake by K-ATP channels in astrocytes, the upregulation of Kir6.2 in AD may be a compensatory effect on its glutamate uptake (Griffith et al., 2016).

**FIGURE 1:**
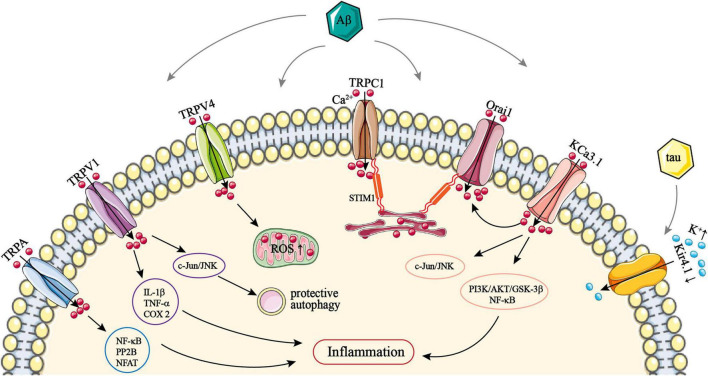
The role of astrocytic TRPA, TRPC1, TRPV1/4, Orai1, KCa3.1, and Kir4.1 channels in AD. TRPA, TRPV1, and TRPV4 channels are all activated by Aβ and thus mediate Ca^2+^ influx, resulting in intracellular Ca^2+^ overload. Ca^2+^ entry *via* TRPA can activate NF-κB, PP2B, and NFAT. Ca^2+^ influx *via* TRPV1 can upregulate the expression of IL-1β, TNF-α, and COX-2. All of these can promote astrocytic inflammatory responses. Activation of c-Jun/JNK by Ca^2+^ *via* TRPV1 can induce protective autophagy in astrocytes. Ca^2+^ entry *via* TRPV4 can lead to ROS production. STIM1 on the astrocytic ER membrane recruits TRPC1 trafficking vesicles anchored to the plasma membrane, and mediating Ca^2+^ influx *via* a STIM1–TRPC1 complex. STIM1 also associates and activates Orai1; this also facilitates Ca^2+^ entry *via* a STIM1–Orai1 complex. Both TRPC1 and Orai1 can be upregulated by Aβ, which promotes SOCE-mediated Ca^2+^ influx. Astrocytic KCa3.1 channels can activate Orai1, and then also facilitate Ca^2+^ entry *via* the STIM1–Orai1 complex. KCa3.1 can also mediate Ca^2+^ influx directly and subsequently activate PI3K/AKT/GSK-3β, NF-κB, and c-Jun/JNK pathways, which promote astrocyte hyperactivation. Kir4.1 channels mediate K^+^ influx; Aβ and tau can downregulate astrocytic Kir4.1, which leads to an excessive extracellular K^+^ concentration. AD, Alzheimer’s disease; Aβ, amyloid-β; AKT, protein kinase B; COX-2, cyclooxygenase 2; ER, endoplasmic reticulum; GSK-3β, glycogen synthase kinase 3; IL-1β, interleukin-1β; JNK, c-Jun N-terminal kinase; Orai1, Ca^2+^ release-activated Ca^2+^ channel protein 1; NF-κB, nuclear factor κB; NFAT, nuclear factor of activated T-cells; PI3K, phosphoinositide 3-kinase; PP2B, serine/threonine-protein phosphatase 2B; ROS, reactive oxygen species; SOCE, store-operated Ca^2+^ entry; STIM1, stromal interacting molecule 1; TNF-α, tumor necrosis factor α; TRP, transient receptor potential; TRPA, TRP ankyrin; TRPV, TRP vanilloid.

Among the members of the Kir channel family, Kir4.1 in astrocytes also plays important roles in the homeostasis of the CNS, maintaining K^+^ homeostasis and resting membrane potential, and facilitating glutamate uptake. Although Kir4.1 channels are only found in glial cells, the abnormal function of this channel can cause neuronal hyperexcitability and is associated with neurodegenerative diseases, including AD (Nwaobi et al., 2016). A postmortem study indicated that reduced Kir4.1 expression in patients with AD exhibited moderate to severe CAA. In mice with varying degrees of CAA, a decrease in Kir4.1 expression also occurred in astrocytes in their neurovascular units (Wilcock et al., 2009). Furthermore, pentylenetetrazole (an inducer of epilepsy)-treated Fyn [an Src family kinase which can be activated by Aβ and interacts with tau, co-localizing with tau in the hippocampus of AD (Nisbet and Götz, 2018)] KO, tau KO, and double KO mice exhibit less inhibition of Kir4.1 in astrocytes compared to the control group as well as reduced gliosis and neurodegeneration (Figure 1) (Putra et al., 2020). In addition, the loss of astrocyte Kir4.1 in pathological states is similar to the loss of AQP4; both anchor to DP71 in astrocyte terminal protrusions. In vascular cognitive impairment and dementia (often coexisting with AD, the second leading cause of dementia after AD) mouse models, Sudduth et al. (2017) demonstrated the destruction of astrocyte terminal protrusions and a decrease in DP71, AQP4, and Kir4.1 localization along with neuroinflammation and cognitive dysfunction. In conclusion, the decrease in Kir4.1 expression and loss of DP71 due to structural abnormalities in astrocytes may both be common causes of Kir4.1 reduction in astrocytes in AD. Furthermore, Nwaobi et al. (2014) identified the epigenetic regulation of Kir4.1 channels in rats. DNA methylation levels of the Kir4.1 gene, *KCNJ10*, were negatively correlated with Kir4.1 expression in astrocytes, which implies that epigenetic regulation of Kir4.1 might be a possible therapeutic direction for neurodegenerative diseases associated with Kir4.1 channels, including AD.

## Astrocytic ion Channels in Parkinson’s Disease

### Astrocytic Aquaporins in Parkinson’s Disease

The loss of dopaminergic neurons in the substantia nigra compact area and the aggregation of Lewy bodies and Lewy neurites are the main features of Parkinson’s disease (PD). α-SYN is one of the main components of Lewy bodies (Rocha et al., 2018), and its gene (mutant α-*SYN*) is pivotal in familial PD (Tang and Le, 2016). When α-SYN folds into oligomers by mistake or has modifications, such as phosphorylation, ubiquitination, and nitrification, it will exert neurotoxic effects (Zhang et al., 2017; Riederer et al., 2019). Astrocytes can detect and clear α-SYN. It was shown that blocking brain lymphatic drainage in A53T transgenic mice (capable of expressing increased α-SYN) disrupted CSF–ISF exchange and impaired AQP4 polarization in the substantia nigra, subsequently exacerbating α-SYN aggregation and leading to impaired dopaminergic neurons (Zou W. et al., 2019). Mouse models of stroke exhibited reduced expression and abnormal localization of AQP4 channels on the astrocyte end-foot process, which led to an increase in the aggregation of α-SYN (Sanchez-Bezanilla et al., 2019). A postmortem study found that the levels of AQP1 and AQP4 in the astrocytes of patients with PD negatively correlated with α-SYN deposition (Hoshi et al., 2017). In addition, investigating the effect of AQP4 on α-SYN expression in the CNS, Xue et al. (2019) found that in astrocytes, the presence of AQP4 reduced α-SYN mRNA expression; however, α-SYN was not evident at the protein level. Thus, the clearance of α-SYN is likely to involve AQPs on astrocytes, especially AQP4. Interestingly, in astrocyte terminal protrusions, α-SYN is also an anchor protein of AQP4, implying that deletion of α-SYN affects the localization of AQP4. Regarding the extent of the effect of α-SYN on the water permeability of the membranes of brain gray matter cells, no significant difference was found in cortical intermediate metabolites between α-SYN–deficient and AQP4-deficient mice. However, the effect of α-SYN deficiency on the distribution of AQP4 channels affected brain glucose metabolism (Pavlin et al., 2017).

The role of AQPs in the pathogenesis of PD goes beyond their interaction with α-SYN. Transforming growth factor (TGF)-β1 is mainly derived from astrocytes and microglia, which restrains neuroinflammation and has a neuroprotective effect. In the presence of 1-methyl-4-phenyl-1,2,3,6-tetrahydropyridine (MPTP; used to induce PD symptoms in animal models), AQP4-KO astrocytes failed to upregulate TGF-β1 expression (Xue et al., 2019). Glial cell line–derived neurotrophic factor (GDNF) can nourish dopaminergic neurons and maintain neuronal survival in an environment of oxidative stress and neuroinflammation (Yue et al., 2018). Regarding the relationship between AQP4 and GDNF generation, a study found that GDNF production in astrocytes was downregulated in AQP4-deficient mice (Fan et al., 2008). The astrocytic AQP4 channel also shows an inhibitory effect on the inflammatory pathway. Sun et al. (2016) indicated that an IKB kinase (IKK)/NF-κB pathway led to ATP generation as well as the upregulation of TNF-α and IL-1β in midbrain astrocytes in AQP4-deficient mice. In addition, in a co-culture of astrocytes and microglia exposed to methylphenylpyridine (MPP^+^; an active and toxic metabolite of MPTP; also causes the manifestation of PD in animal models), the loss of astrocytic AQP4 exacerbated the activation of microglia. This might be because of the release of pro-inflammatory cytokines from reactive astrocytes. What is more, it has been shown that dopaminergic neurons in the substantia nigra and ventral tegmental area differ in their response to MPTP. However, the difference in the susceptibility of dopaminergic neurons in these two different brain regions disappears in a mouse model of AQP4 deficiency. AQP4-deficient mice were more likely to show neurotoxicity in response to MPTP with more reactive astrocytes and microglia in both the substantia nigra and ventral tegmental areas (Zhang et al., 2016). Overall, these results demonstrate the inhibitory effect of AQP4 channels in astrocytes on neuroinflammation in PD models. However, evidence exists that AQP4-mediated astrocyte swelling also has a role in promoting neuroinflammation. Prydz et al. (2020) also injected MPP^+^ into the striatum of AQP4-deficient mice. Wild-type mice were found to have more pronounced neuroinflammation than AQP4-KO mice and a deficiency of astrocytic AQP4 channels induced weaker microglia activation than in wild-type mice. The above two studies used similar methods to find that AQP4 mediates the interaction between astrocytes and microglia; however, the two articles report opposing AQP4 activities. In addition, the latter study also revealed an upregulation of astrocyte AQP4 expression by MPP^+^ in the substantia nigra of mice, which also caused swelling of their astrocytic terminal protrusions. The study also analyzed the specific reasons for this, suggesting that the absence of AQP4 reduces the swelling of the astrocytic end-foot processes, thereby decreasing the release of pro-inflammatory factors and substances that activate microglia caused by swelling. Meanwhile, the swelling of astrocytes with AQP4 deficiency was much less severe. Also, the study concluded that the reason for these opposing findings lay in the integrity or otherwise of the BBB in mice. In contrast to the intact BBB in this study, disruption of the BBB can expose neurons and glial cells directly to inflammatory cells and factors (Prydz et al., 2020). The swelling effect in AQP4-induced astrocytes appeared to also be observed in the brain tissue of patients with PD, who show more severe water accumulation in the substantia nigra. Higher levels of AQP4 in nigrostriatal astrocytes were also found in a comparative study of mouse cortical and substantia nigra; the substantia nigra was also more prone to fluid accumulation (Prydz et al., 2017).

AQP9 channels are another member of the AQP family and are also present in astrocytes. Unlike the aforementioned AQP1 and 4 channels, AQP9 channels are not only permeable to water, lactate, and glycerol, but also several other solutes (Hirt et al., 2018). Examining the subcellular distribution of AQP9 in the CNS, it was found that this was selectively expressed in the inner mitochondrial membrane (IMM) of rat dopaminergic neurons and astrocytes. In view of the location of AQP9 in the IMM and its permeability to lactic acid, AQP9 confers cells with resistance to ischemia (Amiry-Moghaddam et al., 2005). However, the permeability of AQP9 to molecules other than water is a “double-edged sword.” In PD, AQP9 can allow environmental toxins to enter the mitochondria. Stahl et al. (2018) found that MPP^+^-mediated neurotoxicity was associated with the diffusion of MPP^+^ through AQP9 in mouse models. In response to MPP^+^, nigrostriatal dopaminergic neurons were less damaged in AQP9-deficient mice. In addition, this study showed that AQP4 is not permeable to MPP^+^. For astrocytes, mice overexpressing AQP9 showed more pronounced astrogliosis, while astrocytes with Ser222 phosphorylated AQP9 showed more pronounced proliferation as described above (Hirt et al., 2018). These findings suggest that the permeability of AQP9 to substances plays an important role in both the physiological and pathological manifestations of the CNS. Therefore, how to regulate this “door” in the IMM may have potential implications for the treatment of PD. In addition, studies on the role of AQP9 in PD have mainly focused on neurons, and the mechanisms of AQP9-mediated astrocyte proliferation in PD may be a future research direction.

### Astrocytic Transient Receptor Potential Vanilloid Channels in Parkinson’s Disease

In neurodegenerative diseases, the neuroprotective role of TRPV1 channels in astrocytes does not solely lie in their mediated autophagy as mentioned previously. Ciliary neurotrophic factor (CNTF) is an important motor neuron trophic factor that promotes neuronal survival and has a potential therapeutic role in neurodegenerative diseases (Bongioanni et al., 2004). Coincidentally, CNTF production by astrocytes and the neuroprotective effects of this factor are associated with TRPV1 channels. In α-SYN overexpression and MPP^+^-treated mouse models, TRPV1 channels in astrocytes promoted CNTF production and thus inhibited dopaminergic neuron degeneration (Nam et al., 2015). A postmortem study also showed that levels of TRPV1, CNTF, and GFAP (a marker of astrogliosis) were significantly higher in the substantia nigra of patients with PD than controls, as well as in GFAP^+^ astrocytes of patients with PD; a significant difference in the expression of these proteins in the cortex was not noted (Nam et al., 2015). Baek et al. (2018) not only confirmed the promotion of CNTF produced by TRPV1 in astrocytes, but also found that the above process as induced by capsaicin, a TRPV1 agonist, effectively inhibited microglial activation and oxidative stress in the substantia nigra of MPP^+^-treated rats. In addition, cannabidiol also promoted TRPV1-mediated CNTF production in astrocytes, subsequently exerting neuroprotective effects (Giuliano et al., 2021). This therapeutic effect of capsaicin was also seen in nigrostriatal dopaminergic neurons and microglia (Chung et al., 2017), which increased their tyrosine hydroxylase activity and then promoted the release of dopamine (Kim et al., 2019). Therefore, the promotion of endogenous CNTF in astrocytes by TRPV1 channels can significantly reduce neurotoxicity, highlighting a new role for TRPV1 channels in the pharmacological treatment of PD.

### Astrocytic ATP-Sensitive Potassium and Kir Channels in Parkinson’s Disease

In the CNS, excess glutamate leads to neurotoxicity, one of the main mechanisms of PD, which is mainly related to the impaired uptake of glutamate by glial cells (Iovino et al., 2020). K-ATP channels are one of the key structures for glutamate uptake by astrocytes. With regard to the role of open K-ATP channels in PD, the K-ATP channel opening inducer, iptakalim, was found to reduce glutamate levels and increase dopamine concentrations in the striatum of 6-hydroxydopamine-lesioned PD rat models as well as reverse the decline in glutamate uptake in astrocytes induced by 6-hydroxydopamine (Wang et al., 2006). In addition, an *in vitro* study revealed the facilitative effect of iptakalim on glutamate uptake by astrocytes exposed to MPP^+^ (Hu et al., 2005). Therefore, K-ATP channels in astrocytes promote glutamate uptake as a neuroprotective effect in PD.

The role of K-ATP in maintaining astrocytic gap junctions was mentioned earlier, and this has also been studied in PD. Abnormalities in gap junction intercellular communication can lead to the entry of water into the parenchyma through the BBB, affecting ion transport in astrocytes and causing astrocyte swelling, as well as affecting glutamatergic gliotransmission, which is implicated in the pathogenesis of PD (Mesnil et al., 2020). Rotenone is used to induce PD in animal models and inhibits the expression of astrocytic connexin 43 (a major protein that forms astrocyte gap junctions) and reduces the permeability of gap junctions. Zhang et al. (2011) showed that the use of iptakalim and diazoxide to promote the opening of K-ATP channels in astrocytes effectively inhibited the rotenone-induced decrease in connexin 43 levels in astrocytes, improved the permeability of gap junctions, and reduced astrocyte apoptosis. Therefore, improving the gap junctions of astrocytes may be a new strategy for PD treatment, and K-ATP channels are the entry point for this strategy.

Mitochondrial dysfunction plays a pivotal role in the pathogenesis of PD, increasing the production of ROS that triggers oxidative stress, and interacts with α-SYN to exacerbate the pathological manifestations of PD. This has been considered as an early event in PD and has become a hot topic of research into PD treatment (Grünewald et al., 2019; Wang et al., 2021). In recent years, the regulation of these mechanisms in PD by K-ATP channels in astrocytes has been shown. Hu et al. (2019) indicated that mitophagy was impaired in Kir6.1-deficient astrocytes of mice, which resulted in the accumulation of excess damaged mitochondria that could not be cleared by autophagy as evidenced by the downregulation of microtubule-associated protein light chain 3-II (LC3-II) and PTEN-induced putative kinase 1 (PINK1)/parkin, thus producing increased ROS and promoting neuroinflammation. Moreover, the substantia nigra compacta (SNc) of Kir6.1-deficient mice exhibited excessive astrogliosis, NLRP3-mediated neuroinflammation, and downregulation of dopamine levels, which could be alleviated by improved mitophagy. In addition, the induction of K-ATP channel openings in astrocytes by iptakalim inhibited mitochondrial apoptosis by inhibiting glutathione depletion. This prevented the loss of a difference in mitochondrial membrane potential and inhibited the production of pro-apoptotic factors, such as mitochondrial cytochrome C and apoptosis-inducing factor in astrocytes, as well as activated the ERK/MAPK pathway and inhibited JNK phosphorylation to inhibit astrocyte apoptosis induced by MPP^+^ (Zhang et al., 2007). In addition, iptakalim also attenuated the hyperactivation of astrocytes and microglia in the nigrostriatal densities of MPP^+^-treated mice, inhibited the activation of astrocyte p38MAPK and the release of TNF-α, and improved the locomotor behavior of mice. All of these effects were inhibited by the mitochondrial K-ATP channel blocker 5-hydroxydecanoate (Yang et al., 2009). Thus, the regulation of mitochondrial function by K-ATP channels in astrocytes shows a wide range of roles in the CNS, including attenuation of oxidative stress, apoptosis, astrocyte hyperactivation, and neuroinflammation. It also reflects that promoters of K-ATP channel opening, such as iptakalim, have promising prospects for the treatment of PD.

Moreover, Kir6.1 subunits have properties that regulate astrocytic complement activation. A study in mice of LPS-induced PD demonstrated that deletion of astrocyte Kir6.1 activated NF-κB thereby promoting the production of complement C3. Complement C3 then acted on neuronal C3a anaphylatoxin receptor (C3aR) to cause neuronal death in the SNc. This reflects the neurotoxicity produced by the complement activation pathway in astrocytes. Additionally, inhibition of NF-κB in astrocytes or blockade of C3aR in neurons effectively reduced nerve damage (Chen et al., 2021). This suggests that the inhibitory effect of astrocytic Kir6.1 channels on the astrocytic complement pathway is a novel pathway for the neuroprotective role of K-ATP channels in PD.

### Astrocytic P2X7R in Parkinson’s Disease

**FIGURE 2:**
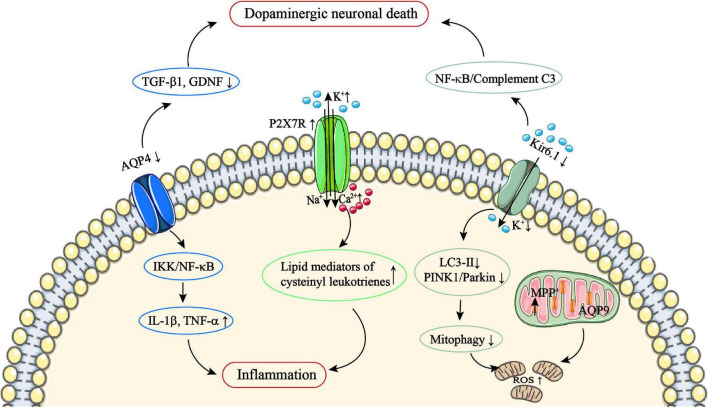
The role of astrocytic AQP4/9, P2X7R, and Kir6.1 channels in PD. AQP4 deficiency fails to upregulate TGF-β1 and GDNF expression, and upregulates the IKK/NF-κB pathway, which subsequently facilitates TNF-α and IL-1β production and results in an inflammatory response. AQP9 channels are located on the IMM and allow environmental toxins such as MPP^+^ to enter the mitochondria. In addition, Kir6.1 deficiency fails to upregulate LC3-II and PINK1/Parkin, leading to impairment mitophagy, which can result in excessive damage of the mitochondria, and subsequently lead to ROS production. Kir6.1 deficiency can also lead to NF-κB activation and subsequently facilitate complement C3 production. Complement C3 then acts on neuronal C3aR to cause dopaminergic neuronal death. Astrocytic P2X7R overexpression can result in excessive Ca^2+^ influx; this can upregulate the level of lipid mediators of cysteinyl leukotrienes and subsequently lead to inflammation. AQP, aquaporin; C3aR, C3a anaphylatoxin receptor; GDNF, glial cell line–derived neurotrophic factor; IKK, IKB kinase; IL-1β, interleukin-1β; IMM, inner mitochondrial membrane; MPP^+^, methylphenylpyridine; NF-κB, nuclear factor κB; P2X7R, P2X7 receptor; PD, Parkinson’s disease; PINK1, PTEN-induced putative kinase 1; ROS, reactive oxygen species; TGF-β1, transforming growth factor β1; TNF-α, tumor necrosis factor α.

In the CNS, P2X7R is preferentially present in microglia, and its mediated inflammatory response is involved in the pathogenesis of PD. In rat models of acute PD, P2X7R binding was increased in the striatum and co-localized with microglia (Crabbé et al., 2019; Illes, 2020). Although P2X7 is not as well distributed in astrocytes as in microglia, P2X7R-mediated transmembrane transport of Ca^2+^ and K^+^ also take part in the activation of astrocytes, which lead to neuroinflammation (Illes, 2020; Zhao et al., 2021). It has been confirmed that stimulating astrocytic P2X7R can promote the production of glutamate and NO, and excessive Ca^2+^ influx *via* P2X7R can also upregulate the level of lipid mediators of cysteinyl leukotrienes that can lead to inflammation (Figure 2) (Sidoryk-Węgrzynowicz and Strużyńska, 2021). To investigate the expression of P2X7R in astrocytes in PD, Gao et al. (2011) demonstrated that low concentrations of rotenone (2–20 nM) increased the current density of P2X7R in rat astrocytes, which down-regulated the secretion of TNF-α in astrocytes, while the expression of P2X7R did not change significantly). In addition, blocking P2X7R exhibited ameliorative effects on PD. Antagonizing P2X7R with Brilliant Blue G effectively inhibited astrogliosis and microgliosis in the striatum, improved synaptic function, and increased dopamine levels in the substantia nigra and striatum of rats in 6-hydroxydopamine PD models (Carmo et al., 2014). Similarly, Fonteles et al. (2020) reached conclusions consistent with the above study. The blocking effect of Brilliant Blue G on P2X7R inhibited the neuroinflammation mediated by astrocytes and microglia in 6-hydroxydopamine rats, in addition to alleviated dopamine-induced behavioral symptoms and balanced purine signaling. Moreover, the activation of P2X7R showed a negative correlation with AQP4 expression in mouse astrocytes; this may due to inhibition of AQP4 by Ca^2+^-dependent protein kinase C (Lee et al., 2008). Although the density of P2X7R in astrocytes is lower than that of microglia (Zhao et al., 2021), blocking P2X7R in astrocytes can reduce astrocytic activation and neurotoxicity, which has potential implications for the treatment of PD.

## Astrocytic ion Channels in Amyotrophic Lateral Sclerosis

### Astrocytic Aquaporin 4 in Amyotrophic Lateral Sclerosis

As a neurodegenerative disease affecting motor neurons, motor neuron damage and dysfunction in amyotrophic lateral sclerosis (ALS) are associated with astrogliosis changes in the astrocytic environment, and ALS is also accompanied by dysfunction of the BBB (Zou S. et al., 2019). Since astrocytic terminal protrusions ensheath the wall of cerebral blood vessels to maintain the normal function of the BBB (Haj-Yasein et al., 2011), and astrocytic terminal protrusions have a large amount of AQP4 channels that participate in maintaining the integrity of the BBB, studies have attempted to elucidate the pathogenesis of ALS by focusing on AQP4 channels of astrocytes (Zou S. et al., 2019). Increased astrocytic AQP4 expression has been shown in the brainstem, cortex, and around spinal cord capillaries of rats in ALS models (Nicaise et al., 2009; Bataveljić et al., 2012). Excessive levels of AQP4 also induced destruction of the BBB in mouse models of ischemia (Tang et al., 2014). With regard to the role of astrocytic AQP4 on the BBB in ALS, Watanabe-Matsumoto et al. (2018) not only demonstrated the overexpression of AQP4 in astrocytes of mice and patients with ALS, but also found an improvement in the permeability of the BBB in ALS mice deficient in AQP4. What is more, it has been suggested that disruption of the BBB can induce astrocytic AQP4 expression. Therefore, AQP4 overexpression in astrocytes and BBB dysfunction in ALS may be reciprocal (Tomás-Camardiel et al., 2005; Watanabe-Matsumoto et al., 2018). In fact, the AQP4 of astrocytes in ALS is not only overexpressed, but the localization of AQP4 channels is also abnormal. In superoxide dismutase (SOD) mutant ALS mice, the overall level of astrocytic AQP4 was increased in the ventricornu, while AQP4 polarization in astrocytes was reduced as a result of localization from the terminal protrusions of swollen astrocytes to the cell membrane (Dai et al., 2017). Therefore, the relationship between the level of astrocytic AQP4 and BBB function as well as the polarization of AQP4 channels in astrocytes affect the progression of ALS.

### Astrocytic Kir Channels in Amyotrophic Lateral Sclerosis

**FIGURE 3:**
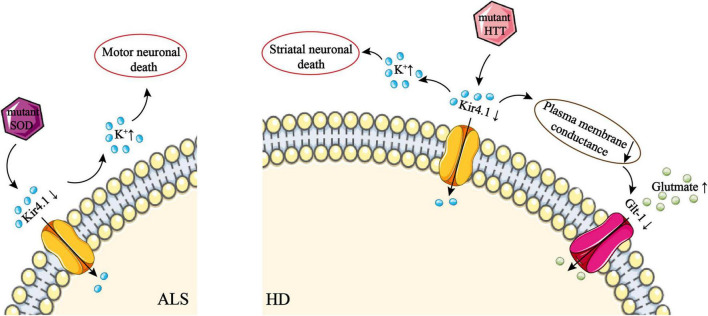
The role of Kir4.1 channel deficiency in ALS and HD. Downregulation of Kir4.1 by mutant SOD disrupts K^+^ influx, which leads to an excessive extracellular K^+^ concentration. This disrupts the extracellular environment of neurons and subsequently causes motor neuron death. In addition, mislocalization and overexpression of AQP4 channels on astrocytes can lead to astrocyte swelling. This may downregulate AQP4 due to the sensitivity of AQP4 to stretch, although this mechanism is uncertain. Downregulation of Kir4.1 by mutant HTT disrupts K^+^ influx, which leads to an excessive extracellular K^+^ concentration. This disrupts the extracellular environment of neurons and subsequently causes striatal neuron death. High conductance of the plasma membrane *via* Kir4.1 can activate Glt-1, which takes part in the uptake of glutamate by astrocytes. However, downregulation of Kir4.1 leads to a decrease in plasma membrane conductance, which disrupts glutamate uptake by astrocytes. ALS, amyotrophic lateral sclerosis; AQP, aquaporin; Glt-1, glial glutamate transporter 1; HD, Huntington’s disease; HTT, huntingtin; SOD, superoxide dismutase.

Motor neurons are very sensitive to K^+^ and excessive extracellular concentrations of K^+^ can cause motor neuron death, which leads to ALS symptoms. As early as 15 years ago, a study revealed the loss of Kir4.1 channels in spinal cord extracts from SOD mutant ALS mice, suggesting a link between Kir4.1 channels and neuronal damage in ALS (Kaiser et al., 2006). A subsequent study found not only significant astrogliosis with reduced Kir4.1 channels in the trigeminal nucleus of patients with ALS, but also a decrease in Kir4.1 protein levels and current density in rat cortical astrocytes of an ALS group. In addition, inhibition of Kir4.1 currents can lead to high extracellular K^+^ concentrations, reflecting the reduced K^+^ buffering potential of astrocytes in ALS, and excessive extracellular K^+^ concentrations can cause motor neuron death (Bataveljić et al., 2012). Furthermore, Kelley et al. (2018) demonstrated a downregulation of Kir4.1 at transcription and translation levels in astrocytes *in vitro* from SOD1 mutant ALS patients, which reinforced evidence on astrocytic Kir4.1 deficiency and the pathogenesis of ALS (Figure 3). Therefore, these demonstrate the role of astrocytic Kir4.1 channels in regulating extracellular K^+^ concentrations in the pathogenesis of ALS. This does not appear to be a unique mechanism by which abnormalities in astrocytic Kir4.1 channels are involved in neuronal damage in ALS. Regulation of K^+^ concentration by Kir4.1 and the passage of water *via* AQP4 on astrocytic terminal protrusions work together to maintain the equilibrium of intra- and extracellular osmotic pressure (Peric et al., 2017). However, Kir4.1 channels are sensitive to stretch and evidence suggests that AQP4 channel-mediated cell swelling affects Kir4.1 currents (Nagelhus and Ottersen, 2013). The down-regulation of Kir4.1 channels and up-regulation of AQP4 showed strong synchronicity in astrocytes of SOD mutant ALS rats, which seems to indicate a possible interaction between these two ion channels (Bataveljić et al., 2012). Meanwhile, with regard to the terminal protrusions of astrocytes, both AQP4 and Kir4.1 channels are anchored to the same protein, DP71 (Sudduth et al., 2017). However, few reports exist on the expression of DP71 in ALS. Therefore, the subtle relationship between Kir4.1 and AQP4 channels in the molecular mechanism of ALS needs to be further investigated (Bataveljić et al., 2012).

## Astrocytic Kir Channels in Huntington’s Disease

Huntington’s disease (HD) is characterized by progressive degeneration of striatal neurons with mutant huntingtin (mHTT)-encoded proteins that induce striatal neuron death. Evidence suggests that patients with HD and animal models exhibit an accumulation of mHTT in astrocytes that is thought to be associated with a decrease in astrocytic Kir4.1 channel levels (Nwaobi et al., 2016). In a R6/2 HD mouse model, the level of Kir4.1 channels decreased in its striatal astrocytes, showing abnormal astrocytic electrophysiology and an increased concentration of extracellular K^+^. Upregulating Kir4.1 expression improved these findings, and reduced neural damage and HD-like motor behavior (Khakh and Sofroniew, 2014). In addition, increasing the concentration of K^+^ in the brain tissue of a normal mouse also caused pathological manifestations similar to those in a HD group, suggesting that impairment of Kir4.1 channels in astrocytes in HD can lead to the abnormal transport and distribution of K^+^, which caused neuronal dysfunction (Khakh and Sofroniew, 2014). The promotion of glutamate uptake in astrocytes by Kir4.1 was mentioned earlier, and this is also found in HD. Down-regulation of the expression of the glutamate transporter, glial glutamate transporter 1 (Glt-1), occurs in astrocytes of PD mouse models, while in R6/2 and Q175 HD mice, an increase of Kir4.1 in astrocytes enhanced the activity of Glt-1 by increasing the conductance of the plasma membrane, which promoted the uptake of glutamate by astrocytes and improved spontaneous astrocytic Ca^2+^ signals (Figure 3) (Tong et al., 2014; Dvorzhak et al., 2016; Jiang et al., 2016). In addition, intravenous injection of adeno-associated viral vector-Kir4.1-enhanced green fluorescent protein into Q175HD mice repolarized their astrocytes and reduced astrocytic spontaneous action potentials, and also improved the motor behavior of HD mice. This further enriched the therapeutic effect of Kir4.1 on HD (Vagner et al., 2016). What is more, Tong et al. (2014) demonstrated an inhibitory effect of mHTT on Kir4.1 expression in striatal astrocytes of R6/2 HD mice, which provided a new direction for the study of HD mechanisms. In summary, the promotion of Kir4.1 channels in astrocytes for the uptake of glutamate and the effect of transporting ions on stabilizing the membrane potential reflect the important role of its deletion in HD. In addition, continued exploration of the effect of mHTT on Kir4.1 will help to further improve research on the pathogenesis of HD.

## Conclusion and Future Perspectives

Reactive astrogliosis, which can lead to neuroinflammation, is widespread in neurodegenerative diseases. Ionic disturbances in astrocytes, particularly Ca^2+^ overload, play an important role in this process. Abnormalities in the expression, localization, and function of ion channels can cause ion disorders in astrocytes (Table 1). Meanwhile characteristic proteins of neurodegenerative diseases, pro-inflammatory factors, and ROS contribution greatly to ion channel abnormalities.

**TABLE 1:** Pathological manifestations of astrocytic ion channels in neurodegenerative diseases.

Astrocytic ion channels	Diseases	Pathological behaviors	Outcomes	References
AQP4 channels	AD	downregulation of DP71 dystrophin by Aβ	AQP4 mislocalization	Wilcock et al., 2009; Eide and Hansson, 2018
		mislocalization	accumulation of Aβ and tau (decrease of Aβ and tau clearance)	Iliff et al., 2012; Iliff et al., 2014; Boespflug et al., 2018; Harrison et al., 2020; Reeves et al., 2020
	PD	deficiency	accumulation of α-SYN	Hoshi et al., 2017; Sanchez-Bezanilla et al., 2019; Zou W. et al., 2019
		deficiency	upregulating TNF-α and IL-1β *via* IKK/NF-κB pathway	Sun et al., 2016
	ALS	overexpression and altered subcellular localization	BBB disruption	Amiry-Moghaddam et al., 2003; Tang et al., 2014; Dai et al., 2017; Watanabe-Matsumoto et al., 2018; Glober et al., 2019
		BBB disruption	astrocytic AQP4 overexpression	Tomás-Camardiel et al., 2005; Watanabe-Matsumoto et al., 2018
AQP9 channels	PD	allowing environmental toxins including MPP^+^ to enter the mitochondria		Hirt et al., 2018; Stahl et al., 2018
TRPA1 channels	AD	activation by Aβ	excessive Ca^2+^ influx (result in 1. inflammatory responses *via* activating NF-κB, PP2B and NFAT; 2. astrocytes activation)	Lee et al., 2016; Bosson et al., 2017; Paumier et al., 2021
TRPC channels		activation by Aβ	facilitating SOCE	Linde et al., 2011; Ronco et al., 2014
TRPV channels		activation of TRPV4 channels by Aβ	excessive Ca^2+^ influx and ROS production	Bai and Lipski, 2014
		inhibition of TRPV1 channels	suppressing autophagy and inducing astrocytic apoptosis	Liu et al., 2013
Orai1		activation by Aβ	facilitating SOCE	Linde et al., 2011
KCa3.1 channels		activation by Aβ	excessive Ca^2+^ influx	Yi et al., 2016, 2017
		activating Orai1	facilitating SOCE	Yu et al., 2018; Wei et al., 2019
		activating PI3K/AKT/GSK-3β, NF-κB, c-Jun/JNK and ERK1/2 pathway	astrocytes activation	Yu et al., 2017; Wei et al., 2019
K-ATP channels and Kir6	PD	deficiency of Kir6.1	impairment mitophagy due to downregulation of LC3-II and PINK1/Parkin (result in accumulation of excess damaged mitochondria and ROS production)	Hu et al., 2019
		deficiency of Kir6.1	activating NF-κB thereby promoting complement C3 production (acts on neuronal C3aR causing neuronal death)	Chen et al., 2021
Kir4.1 channels	ALS	downregulation by mutant SOD	excessive extracellular K^+^ concentration (result in motor neuronal death)	Kaiser et al., 2006; Bataveljić et al., 2012; Kelley et al., 2018
	HD	downregulation by mutant HTT	excessive extracellular K^+^ concentration (result in neuronal degeneration)	Khakh and Sofroniew, 2014; Tong et al., 2014
		downregulation by mutant HTT	reduced Glt-1 activity due to decrease in plasma membrane conductance (result in decrease in glutamate uptake by astrocytes)	Tong et al., 2014; Dvorzhak et al., 2016; Jiang et al., 2016
P2X7R	PD		excessive Ca^2+^ influx and K^+^ efflux mediated by P2X7R	Illes, 2020; Zhao et al., 2021

*1-methyl-4-phenyl-1,2,3,6-tetrahydropyridine, MPTP; α-synuclein, α-SYN; Alzheimer’s disease, AD; amyloid-β, Aβ; Aβ precursor protein, APP; amyotrophic lateral sclerosis, ALS; aquaporin, AQP; ATP-sensitive potassium, K-ATP; blood–brain barrier, BBB; c-Jun N-terminal kinase, JNK; C3a anaphylatoxin receptor, C3aR; calcium ion, Ca^2+^; calcium release–activated calcium, CRAC; calcium release–activated calcium channel protein 1, Orai1; calcium-activated potassium, KCa; central nervous system, CNS; cerebral amyloid angiopathy, CAA; cerebrospinal fluid, CSF; ciliary neurotrophic factor, CNTF; cyclooxygenase, COX; endoplasmic reticulum, ER; extracellular-signal-regulated kinase, ERK; glial cell line–derived neurotrophic factor, GDNF; glial fibrillary acidic protein, GFAP; glial glutamate transporter 1, Glt-1; glycogen synthase kinase-3β, GSK-3β; huntingtin, HTT; Huntington’s disease, HD; IKB kinase, IKK; inner mitochondrial membrane, IMM; inducible nitric oxide synthase, iNOS; interleukin, IL; interstitial fluid, ISF; inwardly rectifying potassium, Kir; knockout, KO; mammalian target of rapamycin, mTOR; methylphenylpyridine, MPP^+^; microtubule-associated protein light chain 3-II, LC3-II; mutant huntingtin, mHTT; nitric oxide, NO; nuclear factor, NF; nuclear factor of activated T-cells, NFAT; P2X7 receptor, P2X7R; Parkinson’s disease, PD; peroxisome proliferator-activated receptor, PPAR; phosphatidylinositol 3-kinase, PI3K; potassium ion, K^+^; protein kinase B, AKT; PTEN-induced putative kinase 1, PINK1; pyrin domain-containing protein 3, NLRP3; reactive nitrogen species, RNS; reactive oxygen species, ROS; serine/threonine-protein phosphatase 2B, PP2B; sodium ion, Na^+^; store-operated calcium, SOC; store-operated calcium entry, SOCE; stromal interacting molecule 1, STIM1; substantia nigra compacta, SNc; superoxide dismutase, SOD; transforming growth factor, TGF; transient receptor potential, TRP; TRP ankyrin, TRPA; TRP canonical, TRPC; TRP melastatin, TRPM; TRP mucolipin, TRPML; TRP no mechanopotential, TRPN; TRP polycystin, TRPP; TRP vanilloid, TRPV; tumor necrosis factor, TNF.*

Recently, increasing evidence has indicated that the role of ion channels in astrocytes goes beyond the regulation of ion homeostasis. Astrocytic ion-channel–mediated mitophagy and inhibition of pro-apoptotic pathways both reflect the protective effect of ion channels on astrocytes in pathological conditions. Moreover, astrocytic ion channels are able to scavenge characteristic proteins of neurodegenerative diseases, and promote the secretion of neurotrophic factors and the uptake of extracellular glutamate by astrocytes. In addition, the integrity of BBB and the stability of astrocyte gap junctions mediated by astrocytic ion channels were also demonstrated to play neuroprotective roles in astrocytic ion channels in neurodegenerative diseases. Therefore, exploring the role of ion channels in astrocytes will not only improve our understanding of the pathogenesis of neurodegenerative diseases, but will also provide new options for the treatment of these diseases.

## Author Contributions

SW, BW, and XZ made substantial contributions to the conception and design of the study. SW, BW, DS, KZ, XY, and XZ participated in drafting the article. SW and BW created the table and figures. All authors approved the final manuscript.

## Conflict of Interest

The authors declare that the research was conducted in the absence of any commercial or financial relationships that could be construed as a potential conflict of interest.

## Publisher’s Note

All claims expressed in this article are solely those of the authors and do not necessarily represent those of their affiliated organizations, or those of the publisher, the editors and the reviewers. Any product that may be evaluated in this article, or claim that may be made by its manufacturer, is not guaranteed or endorsed by the publisher.
